# Exploring the Therapeutic Effect of Perampanel, a Selective AMPA Receptor Antagonist, on Pathophysiological Changes in hAPP‐J20 Transgenic Alzheimer’s Mice

**DOI:** 10.1002/alz.087875

**Published:** 2025-01-09

**Authors:** Keng Ying Liao, Yue Loong Hsin, Xu Han, Wen‐Ying Chen, Wentai Liu

**Affiliations:** ^1^ Department of Veterinary Medicine, National Chung Hsing University, Taichung Taiwan; ^2^ Department of Neurology, Chung Shan Medical University Hospital, Taichung, Taichung Taiwan; ^3^ Department of Bioengineering, University of California, Los Angeles, CA, USA, Los Angeles, CA USA; ^4^ Department of Veterinary Medicine, National Chung Hsing University, Taichung, Taichung Taiwan; ^5^ UCLA California NanoSystems Institute, University of California, Los Angeles, CA, USA, Los Angeles, CA USA

## Abstract

**Background:**

The initiation of amyloid plaque deposition signifies a crucial stage in Alzheimer’s disease (AD) progression, which often coincides with the disruption of neural circuits and cognitive decline. While the role of excitatory‐inhibitory balance is increasingly recognized in AD pathophysiology, targeted therapies to modulate this balance remain underexplored. This study investigates the effect of perampanel, a selective non‐competitive AMPA receptor antagonist, in modulating neurophysiological changes in hAPP‐J20 transgenic Alzheimer’s mice.

**Method:**

Perampanel was administered to hAPP‐J20 mice and their age‐matched wildtype littermates, aged 20‐25 weeks, during the critical amyloid plaque deposition period in the hippocampus. We continuously monitored the local field potential of the hippocampal CA1 region using long‐term wireless telemetry (sampled at a rate of 2 kHz), capable of calculating rates of high‐frequency oscillations (HFOs) to gauge potential epileptiform activity. To evaluate cognitive function, Morris water maze test was conducted. Glutamatergic pyramidal cells and GABAergic interneurons was quantified to assess neuronal health, and the hippocampal concentration of neuropeptide Y (NPY) was measured as an indirect indicator of mossy fiber sprouting.

**Result:**

Treatment with perampanel resulted in a significant rescue of GABAergic interneuron depletion and reduced the pathological increase of HFOs, indicative of restored excitatory‐inhibitory balance. In line with the restored excitatory‐inhibitory balance, it also attenuated neuronal sprouting labeled by NPY. Additionally, perampanel administration led to a substantial decrease in amyloid plaque accumulation in the hippocampus, though the observed memory deficit in transgenic mice did not change significantly.

**Conclusion:**

Our findings reveal that perampanel, an established antiseizure medication, exerts multifaceted neuroprotective effects in the AD mouse model. The preservation of interneurons, reduction of pathological neural hyperactivity, and decreased amyloid plaque burden highlighted the potential of targeting glutamatergic pathways as a therapeutic strategy. The study warrants further investigation of perampanel as a promising candidate for ameliorating the pathophysiological and cognitive deficits associated with AD.

